# Genomic responses to increased temperature and pollinator selection in *Brassica rapa* L.

**DOI:** 10.1111/nph.70977

**Published:** 2026-02-06

**Authors:** Yanqian Ding, Florian P. Schiestl

**Affiliations:** ^1^ Institute of Systematic and Evolutionary Botany University of Zürich Zollikerstrasse 107 CH‐8008 Zürich Switzerland

**Keywords:** allele frequency shift, biotic–abiotic interaction, experimental evolution, genetic drift, genomic adaptation, thermal stress

## Abstract

- Rapid environmental change reshapes both abiotic stress and biotic interactions, yet it remains unclear how these combined forces structure plants' genomic adaptation. In particular, the joint influence of temperature and pollinator identity, two ecological axes undergoing simultaneous global shifts, has rarely been quantified at genomic resolution.
- We resequenced *Brassica rapa* L. plants after a six‐generation evolution experiment, combining two temperature regimes (ambient vs hot) with three pollination treatments (bumblebee, butterfly, and mixed bumblebee–butterfly), and glasshouse control, to assess how these factors shape genomic responses.
- Using multiple complementary statistics (allele‐frequency trajectories, *F*_ST_ outliers, Cochran–Mantel–Haenszel tests, and local score analyses), we found that adaptive genomic responses differed sharply among pollinators and temperatures: warming strengthened selection in community‐level pollination, yielding the clearest signals in the hot‐generalised treatment; bumblebee pollination showed strong but drift‐obscured genomic change; and butterfly treatments exhibited minimal genomic response.
- Our findings demonstrate that pollinator identity and temperature interact nonadditively to produce distinct, highly context‐dependent adaptive trajectories. This work highlights the importance of accounting for demographic variation and ecological complexity when predicting evolutionary responses to climate‐driven shifts in species interactions.

## 摘要

- 剧烈的气候变化同时影响着非生物以及生物间的互作关系，然而植物基因组如何响应这些因素的协同效应，目前仍不明确。特别是温度和传粉者类型这两个正经历全球性变化的生态轴，它们的联合作用很少在开花植物的基因组上得到量化研究。
- 基于此，我们以油菜*Brassica rapa* L. 人工选育的快速代际品种(两月种子‐种子生长周期)为材料，进行了一项持续六代的演化实验，结合两种温度处理(常温与高温)和三种传粉处理(单一熊蜂传粉、单一蝴蝶传粉以及蝴蝶幼虫的植食性拮抗阶段、熊蜂‐蝴蝶混合传粉+蝴蝶幼虫阶段)，并以温室条件下人工授粉作为对照。随后对所有植株进行了初代以及演化后的全基因组重测序，以量化植物基因组响应不同环境因素的选择压。
- 通过运用多种互补的统计方法(包括起始种群‐演化后种群等位基因频率动态变化、FST离群值分析、Cochran–Mantel–Haenszel检验以及Local‐score分析)，我们发现油菜基因组的适应性响应在不同传粉者和温度处理间存在显著差异:高温强化了混合传粉处理下的选择压力，使得高温‐混和传粉处理组产生了最清晰的基因组信号；单一熊蜂传粉处理表现出全基因组水平强烈的变化，但由于有限的有效种群大小，难以区分遗传漂变的作用，掩盖了大部分的选择压下的基因组信号；而蝴蝶传粉处理则显示出微弱的基因组响应。
- 我们的结果表明，不同的传粉者与温度以非线性叠加的方式相互作用，产生了截然不同且高度依赖于具体情境的适应性演化轨迹。本研究强调，在预测由气候变化驱动的物种间相互作用所引发的适应性演化响应时，必须将有效种群统计变化大小和生态复杂性纳入考量。

## Introduction

Ongoing global environmental change is fundamentally reshaping the selective environments experienced by wild and cultivated plant populations world‐wide. Climate change, including rising temperatures, altered precipitation regimes, and more frequent extreme events, interact synergistically with biological pressures such as changing pollinator communities and herbivore dynamics to create increasingly complex and novel selection landscapes (Cohen & Leach, 2020; Desaint *et al*., 2021). Agricultural intensification at landscape scales, including increased use of agrochemicals and loss of seminatural habitats, represents a major driver of insect declines and community changes, further increasing the evolutionary pressures on plant populations. These combined abiotic and biotic stressors not only affect plant growth and reproduction directly but also modulate key ecological interactions, including pollination and herbivory, with cascading effects on evolutionary trajectories (Anderson & Mitchell‐Olds, 2011; Lasky *et al*., 2014; Zhu, 2016; Sato *et al*., 2024).

The recognition that pollinator‐mediated divergent selection drives floral evolution and plant speciation has deep historical roots, tracing back to Darwin's original insights and formalised by Grant (1949). This foundational concept has evolved into one of the most active areas in plant evolutionary biology, supported by mounting evidence across phylogenetic, ecological, and experimental scales (Fenster *et al*., 2004). Recent advances in experimental evolution methodologies and population genomics now provide unprecedented molecular resolution into these dynamics, revealing how pollinator interactions shape genomic evolution through complex networks of directional, balancing, and frequency‐dependent selection (De‐la‐Cruz *et al*., 2022). The integration of high‐throughput sequencing with controlled evolution experiments has transformed our understanding from phenotype‐focussed studies to genome‐wide portraits of adaptive change.

Distinct pollinator functional groups exert markedly different selection pressures on floral traits such as scent composition, morphological architecture, and nectar production dynamics, driving population‐level divergence in plant reproductive characteristics with remarkable rapidity (Knauer & Schiestl, 2017; Caruso *et al*., 2019; Dorey & Schiestl, 2024). Efficient pollinators such as bumblebees (*Bombus* spp.) can impose strong and consistent directional selection when acting as dominant visitors, generating pronounced allele frequency shifts at loci controlling floral adaptation within surprisingly few generations (Frachon & Schiestl, 2024). This evolutionary potential has been demonstrated through compelling experimental studies: nine generations of bumblebee selection in *Brassica rapa* produced significant trait divergence and altered selfing rates due to pollinator efficiency differences, accompanied by detectable genomic changes at multiple loci (Gervasi & Schiestl, 2017; Kofler *et al*., 2024). Natural populations provide complementary evidence for rapid pollinator‐mediated evolution, revealing how these processes unfold under more complex ecological conditions. Recent integrative studies on monkeyflowers have provided exceptional insights into ongoing adaptive walks during incipient pollinator shifts in wild populations. A 2025 study examined two independent yellow‐flowered forms that arose within red hummingbird‐pollinated species, revealing convergent evolution at multiple biological levels and demonstrating that large‐effect mutations in floral colour genes drive initial pollinator attraction shifts, followed by smaller‐effect changes in floral morphology and scent emission (Wenzell *et al*., 2025). These experimental and field studies collectively reveal that the genetic architecture underlying rapid pollinator‐mediated evolution varies considerably across systems and traits. While some evolutionary transitions are controlled by relatively few major‐effect loci, as seen in *Mimulus* pollinator preferences driven by flower colour and nectar production genes (Bradshaw & Schemske, 2003; Streisfeld & Rausher, 2009), other systems show more polygenic architectures where many small‐effect changes contribute to adaptive shifts (Zan & Carlborg, 2019; Gramlich *et al*., 2022; Minadakis *et al*., 2024). Furthermore, pollination systems involving multiple agent types or dual‐function visitors introduce fundamentally more complex selective regimes that can constrain or redirect evolutionary trajectories. For instance, butterflies function simultaneously as pollinators and herbivores, creating inherently conflicting selection pressures that generate nonadditive and highly context‐dependent evolutionary outcomes (Sletvold *et al*., 2015; Rusman *et al*., 2018, 2020; Schiestl *et al*., 2018; Frachon *et al*., 2023; Figueira *et al*., 2025). Such ecological complexity may be further buffered by phenotypic plasticity and epigenetic modifications, including herbivory‐induced DNA methylation changes that can alter floral traits substantially without requiring genomic sequence evolution (Kellenberger *et al*., 2016, 2018; Mukherjee *et al*., 2019). These mechanisms can effectively decouple phenotypic and genomic responses, significantly complicating the detection of adaptation at the DNA sequence level and highlighting the need for integrated approaches that consider multiple levels of biological pollinator type strongly impacts gene flow patterns both within‐ and among‐plant populations, suggesting the pollinator identity effects extend beyond immediate selection to influence broader evolutionary processes including population connectivity and local adaptation potential.

Temperature represents a critical ecological dimension that directly shapes plant–pollinator interactions by altering both partners' physiology and behaviour. On the plant side, elevated temperatures reduce pollen viability and germination, particularly in crop species (Rojas *et al*., 2024), and affect floral traits that mediate pollinator attraction, such as flower size, anthesis timing, and corolla morphology, potentially excluding mismatched pollinators (Scaven & Rafferty, 2013; Jagtap *et al*., 2023; Traine *et al*., 2024; Rusman *et al*., 2025). These changes cascade through mating systems and gene flow, altering evolutionary trajectories. For instance, heat‐stressed *Vicia faba* populations shift from 17% to 80% outcrossing in field conditions due to changes in pollinator activity and resource allocation, promoting genetic mixing and potentially accelerating selection for heat‐tolerant genotypes (Bishop *et al*., 2017). Earlier anthesis favours early‐foraging species while disadvantaging others, reshaping pollination network structure and cross‐pollination dynamics (Khan *et al*., 2024). Experimental warming studies also demonstrate that plant–bee interactions become less frequent and shorter in duration, ultimately reducing seed quality and quantity due to pollinator‐mediated fitness costs (de Manincor *et al*., 2023). Pollinators, in turn, respond strongly to thermal variation, altering their foraging efficiency, timing, and behaviour in ways that reshape selection pressures on floral traits (Scaven & Rafferty, 2013; Descamps *et al*., 2021; Moss & Evans, 2022). For example, bumblebees under heat stress display reduced flower visitation rates, altered handling times, and modified foraging bout durations, potentially changing both the strength and direction of selection on floral characteristics in nonlinear ways (Walters *et al*., 2022; Naumchik & Youngsteadt, 2023; Gérard *et al*., 2024). These temperature‐induced behavioural changes, in combination with altered plant physiology, may generate feedback loops that modulate the intensity and outcome of adaptive responses.

Beyond these immediate ecological effects, heat stress induces extensive changes at the regulatory, transcriptomic, and epigenetic levels that significantly complicate the detection and interpretation of classical genomic selection signals (Migicovsky *et al*., 2014; Yadav *et al*., 2022). Plants respond to heat stress through complex molecular networks involving signal transduction, metabolite production, and expression of heat stress‐associated genes, with heat stress tolerance being fundamentally polygenic in nature. Recent genome‐wide analyses in Brassicaceae have identified extensive families of heat shock responsive genes, including heat shock proteins (HSPs) and heat shock transcription factors (HSFs), many of which are associated with quantitative trait loci for heat stress response (Cantila *et al*., 2024). These findings suggest that temperature functions not only as a direct selective agent but also as a fundamental modulator of evolutionary potential and the genomic detectability of selection signatures in plant populations.

The interaction between temperature stress and pollinator‐mediated selection creates particularly complex evolutionary scenarios that are increasingly relevant under climate change. Understanding these interactions requires experimental approaches that can disentangle the relative contributions of abiotic and biotic factors while accounting for their nonadditive effects on both phenotypic and genomic evolution. Despite growing recognition of the independent effects of abiotic and biotic factors on plant evolution, their interactive influence on genomic evolution trajectories remains poorly understood (Nawaz *et al*., 2023; Dixit *et al*., 2024). This lack of understanding represents a critical knowledge gap in our ability to predict evolutionary responses under global change scenarios. Here, we address this fundamental gap by experimentally testing how temperature and pollinator identity interact to shape genomic adaptation patterns in a factorial evolution experiment. Using *B. rapa* as a model system, we employed factorial combinations of abiotic conditions (ambient vs hot temperature regimes) and biotic (bumblebee, butterfly, generalised pollinator–herbivore, and hand‐pollinated control) maintained across six generations of experimental evolution. We applied population genomic and functional annotation approaches to identify treatment‐specific selection signatures and investigate how ecological complexity shapes the genomic responses of adaptation. By integrating environmental, ecological, and genomic perspectives, our study provides novel insights into how climate and community‐level interactions jointly govern adaptive trajectories in plants.

## Materials and Methods

### Plant material and experimental evolution design

We used *Brassica rapa* L. (Wisconsin Fast Plants®, Carolina Biological Supply, USA), a mostly outcrossing annual species with a rapid life cycle (*c*. 2 months seed‐to‐seed under glasshouse conditions). This system has been used previously in experimental evolution studies due to its ease of handling, short generation time, and well‐characterised reproductive and genetic features (Gervasi & Schiestl, 2017; Ramos & Schiestl, 2019; Dorey *et al*., 2024; Dorey & Schiestl, 2024). All plant material was produced by previous experimental evolution in Traine (2025), from which we obtained leaf tissue samples for population genomic analysis.

Briefly, the base population consisted of 98 full‐sib families derived from hand‐crosses among 300 parental plants of *B. rapa* Fast Plants to increase standing genetic variation. Experimental evolution was then conducted for six generations in a factorial design, comprising two temperature regimes and four pollination regimes. The temperature regimes were (1) ambient (23°C) and (2) hot (27°C with a weekly 24‐h spike to 30°C), reflecting current and CH2018 projections for mid‐century northern Switzerland (Fischer *et al*., 2022; Federal Office of Meterology and Climatology Meteo Swiss, 2024). All plants were grown in climate‐controlled glasshouse cabins. All pots were watered twice‐daily, with additional checks to maintain equal soil moisture between temperature regimes, minimising any cofounding effects of drought. Within each temperature, we imposed three pollination regimes: bumblebee (*Bombus terrestris*), butterfly (*Pieris rapae*, including herbivory), and generalised (both species; including herbivory). And two control groups from hand‐pollination, served as an abiotic control capturing glasshouse background selection unrelated to pollinator behaviour. Each combination of temperature × pollination regime constituted a treatment, and each treatment was represented by two independent replicate populations (A and B), each initiated with 49 plants (in total 16 populations). Throughout the manuscript, we use ‘regime’ to refer to environmental factors (temperature or pollination), ‘treatment’ for each temperature × pollination combination, and ‘population’ for each replicate evolutionary line.

Bumblebee colonies were obtained from Andermatt Biocontrol (Switzerland) and kept in 3 × 1 × 1 m flight cages with supplemental nectar (BioGluc, Biobest, Westerlo, Belgium) and pollen (Blütenpollen Multiflora, KoRo Handels GmbH, Berlin, Germany). Butterflies were reared from laboratory colonies and supplied with *B. rapa* for feeding and oviposition. They were kept under controlled glasshouse conditions (22 ± 1°C, 60% RH, 16 h : 8 h, light : dark photoperiod) before the pollination. Insect visitation for pollination was allowed within the corresponding temperature regimes: ambient‐evolving plants in the ambient cabin and hot‐evolving plants in the hot cabin so that temperature also directly shaped pollinator activity and foraging behaviour.

Pollination protocols were designed to impose a comparable level of pollen limitation across pollinator types while preserving the natural behavioural differences that generate selection. *Bombus* bumblebees are highly efficient foragers that, under unrestricted conditions, visit nearly all flowers in a cage within a short period. In nature, such saturation rarely occurs because pollen is lost to other insects, grooming, or deposition on nonreproductive surfaces. To recreate a realistic level of pollen limitation in the experimental cages, while maintaining a selective component, we restricted bumblebee access by allowing each individual to visit only three plants before recapture and releasing in total 12 bumblebees sequentially. This approach standardised pollen delivered per bee, but it also meant that only a subset of plants received pollen, which directly reduced the number of reproductive contributors and therefore the realised effective population size ($N_{e}$).

By contrast, whereas bumblebees visit usually all flowers of one plant during a visit, *Pieris* butterflies normally visit fewer flower and more individual plants. Thus, in butterfly treatments, more plants received pollinator visits and thus got pollinated. Because they do not approach saturation even over several days, pollen limitation arises inherently from their behaviour rather than from imposed constraints. Accordingly, 25 mated butterflies were left in cages for 4 d, which yields a similar overall quantity of pollen transfer but typically to a larger number of plant individuals than in the bumblebee treatment. Thus, although pollen limitation *per se* was broadly comparable between pollinator types, the number of plants that actually set seed differed systematically, producing treatment‐specific realised $N_{e}$ values. These demographic differences are crucial for downstream genomic analyses because selection acts on individuals, whereas drift is determined by the number of individuals contributing offspring. For the generalised treatment, the numbers of insects (6 bumblebees, 20 butterflies) were adjusted to maintain comparable total visitation and pollen transfer, while retaining the natural mixture of pollinator and herbivore interactions.

After each generation, seeds were dried and counted; the contribution of each plant to the next generation was proportional to its total seed number, ensuring fitness translates into offspring contribution to the next generation. To reduce maternal and transgenerational effects before genomic sampling, all populations underwent a seventh ‘refresher’ generation grown at ambient temperature with hand‐pollination (10 flowers per plant). Leaf tissues from the first and final generations were collected, immediately frozen at −80°C, and later freeze‐dried (−80°C, 100 h) before DNA extraction. Full experimental details – including insect husbandry, visitation protocols, and trait measurement procedures, are provided in Traine *et al*. (2024, 2025) and in Traine (2025, chapter II).

### DNA extraction and pool‐sequencing

High‐molecular‐weight genomic DNA was extracted from freeze‐dried pooled leaf tissues using sbeadex plant kit in combination with the KingFisher Apex Robot (Thermo Fisher Scientific, Waltham, MA, USA), following the manufacturer's protocol. Pool‐seq libraries were prepared from each evolved population as well as from the ancestral G1 population. Within each pool, equal representation of individual replicates was ensured by first extracting DNA from each plant separately and then combining equal DNA mass per individual within that population. Only individuals with sufficient DNA quality and concentration were retained. An identical DNA amount from each was combined using a Brand pipetting robot, resulting in final pool sizes of 22–30 individuals per population pool (Supporting Information Table S1). Whole‐genome sequencing was performed at BGI Genomics (Poland). Paired‐end libraries were prepared using BGI Optimal DNA Library Prep Kit (BGI, Shenzhen, China) and sequenced on the DNBSEQ‐T7 platform (BGI) with 150‐bp paired‐end reads, targeting an average depth of *c*. 120× per pool to enable accurate allele frequency estimation. Initial read processing was conducted by BGI using SOAPnuke (BGI's in‐house pipeline), with filtering parameters: ‐n 0.001 ‐l 20 ‐q 0.5 ‐‐adaMR 0.25 ‐‐polyX 50 ‐‐minReadLen 150, removing adapters, polyX stretches, and low‐quality reads. After cleaning, reads were assessed with FastQC v.0.12.1, and the first 15 bases were trimmed before alignment.

### Variant calling and filtering

Cleaned reads were aligned to the *Brassica rapa* FPsc v.1.3 reference genome, Phytozome release 12 (https://phytozome.jgi.doe.gov/pz/portal.html) using Bwa‐Mem2 (v.2.2.1, Vasimuddin *et al*., 2019). Variant calling followed the Gatk Best Practices pipeline (Depristo *et al*., 2011; Van der Auwera *et al*., 2013), using HaplotypeCaller for variant detection and Picard (v.3.3.0) to mark and remove duplicate reads (McKenna *et al*., 2010). Indels were separated from single‐nucleotide polymorphisms (SNPs) before downstream filtering (vcftools ‐‐remove‐indels) and were not included in subsequent analysis. Variant recalibration was conducted using a two‐step calibration approach (‐‐filter‐expression ‘QD < 2.0 || FS > 60.0 || MQ < 45.0 || DP > ${maxdepth}’ for SNPs, and ‐‐filter‐expression ‘QD < 2.0 || FS > 200.0’ for indels) (https://bioinformaticsworkbook.org/dataAnalysis/VariantCalling/gatk‐dnaseq‐best‐practices‐workflow.html#gsc.tab=0). Only SNPs located on the 10 chromosomes were retained; unassigned scaffolds were excluded using VCFtools v.0.1.16 (Danecek *et al*., 2011). Additional SNP filtering criteria were defined empirically from the observed read‐depth distribution (mean ≈ 53×; range ≈ 18–160×): ‐‐min‐meanDP 18 ‐‐max‐meanDP 160 ‐‐MAF 0.1 ‐‐min alleles‐2 ‐‐max‐alleles 2 ‐‐max‐missing 0.95 ‐‐minGQ 15. SNPs located in repetitive or low‐complexity regions were excluded using the ‐‐exclude‐bed option with a custom annotation file derived from the *B. rapa* genome. For downstream population genetic analyses, samtools mpileup was used to extract SNP‐level read counts, which were then converted to synchronised allele frequency data using PoPoolation2 (mpileup2sync.pl; Kofler *et al*., 2011b).

### Genetic population structure, differentiation, and diversity

To compare standing genetic variation in Wisconsin Fast Plants with natural *B. rapa* populations, we estimated genome‐wide nucleotide diversity (π_all sites) for the ancestral generation one (G1) population using an all‐sites mpileup including invariant positions (samtools mpileup ‐aa). Analyses were restricted to callable, nonrepetitive regions of the 10 chromosomes using the same repeat mask and depth/quality thresholds applied to SNP filtering (MAPQ ≥ 20, baseQ ≥ 20, min depth 18, max depth 160). π was calculated with PoPoolation (Variance‐sliding.pl; Kofler *et al*., 2011a) and averaged across windows; uncertainty was evaluated via bootstrap resampling.

Allele frequencies for each population were estimated using PoPoolation2 (snp‐frequency‐diff.pl), with filtering thresholds set to ‐‐min‐count 12, ‐‐min‐coverage 18, and ‐‐max‐coverage 150. To assess patterns of within‐population diversity, we calculated nucleotide diversity for all SNPs dataset (π_SNPs), Watterson's θ, and Tajima's *D* using grenedalf (Czech *et al*., 2024), employing the following parameters: ‐‐filter‐sample‐min‐count 2, ‐‐filter‐sample‐min‐read‐depth 4, ‐‐window‐type interval, ‐‐window‐interval‐width 50 000, ‐‐window‐average‐policy valid‐snps, ‐‐filter‐total‐min‐read‐depth 18, and ‐‐filter‐total‐max‐read‐depth 150. Estimates of expected heterozygosity (*H*_e_) were computed using the compute.fstats function from PoolFstat v.2.1.2 (Hivert *et al*., 2018; Gautier *et al*., 2022, 2024), based on the formula H = 1 − Q1, where Q1 represents the probability of identity‐in‐state among alleles within a population. Calculations were based on the filtered set of biallelic SNPs, excluding invariant sites. To evaluate genome‐wide population structure, a principal component analysis (PCA) was performed using the glPca function (adegenet package v.2.1.11, Jombart, 2008; Jombart & Ahmed, 2011), implemented in R v.4.3.1 (R Core Team, 2023). Pairwise population differentiation was estimated using Hudson's *F*_ST_, calculated with the compute.pairwiseFST function in PoolFstat v.2.1.2.

### Drift expectations and simulations

As a consequence of the pollen‐limitation design and random variation in visitation, the number of plants that contributed seeds differed across populations, producing different population‐specific realised effective population sizes (*N*_e_, Table S1). Nonvisited plants were excluded from seed collection to ensure reproductive success reflected realised pollination. In the bumblebee treatments, only the subset of plants visited by bumblebees contributed to the next generation (*N*_e_ ≈ 18). In butterfly and generalised treatments, a larger proportion of plants received sufficient visitation, yielding larger *N*_e_ (≈35). These *N*_e_ estimates were derived empirically from census data and used to parameterise neutral simulations and drift expectations.

We then quantified neutral expectations for allele‐frequency change (|ΔAF|) under the realised demography using:

$$\mathrm{Var}\left(p_{t}\right)=p_{0}\left(1-p_{0}\right)\left[1-\prod_{g=1}^{t}\left(1-\frac{1}{2N_{e,g}}\right)\right]$$

where *p*_0_ is the ancestral allele frequency, *p* is the allele frequency after *t* generations, and *N*_e,*g*_ is the realised effective population size in generation *g*. Each term describes drift in a single generation. Multiplying them links drift across generations. This gives the expected amount of allele‐frequency change under neutrality for each treatment. We used this expression to calculate the neutral range (95% bounds) for |ΔAF|, which were then compared with observed allele‐frequency shifts. To complement the analytical range, we also simulated 10^5^ neutral SNPs per treatment by drawing initial frequencies from the empirical G1 allele‐frequency spectrum and propagating them forward for six generations using the treatment‐specific *N*_e_ sequence (*N*_e1_…*N*_e6_). The resulting null distributions of |ΔAF| and *F*_ST_ were then compared against observed values, and excess tail probability was interpreted as significant support for locus‐specific selection.

To account for background genomic change unrelated to pollinator‐mediated selection, we additionally used the hand‐pollinated populations (ambient and hot) as empirical controls, capturing adaptation to glasshouse conditions but lacking biotic selective agents (CBA/CBB, HBA/HBB).

### Genetic signatures of selection

To identify genomic regions under selection in our experimental evolution study, we implemented a comprehensive analytical framework integrating SNP‐level outlier tests, regional signal enrichment, and cross‐method validation. Each treatment was compared against a shared initial (G1) pool. Given the short evolutionary time frame and the pool‐sequencing approach, our pipeline was designed to maximise detection sensitivity while controlling for false positives and replicate effects. Here, we note a key methodological comparison with earlier bumblebee‐selection experiments conducted in the same system. Previous studies (Dorey *et al*., 2024) used individually sequenced plants and phenotype–genotype association tests, which provide high sensitivity for detecting selection even when drift is substantial. By contrast, our pool‐seq does not link genotypes to individual fitness components, drift must be modelled explicitly, and detection thresholds must be more conservative to avoid false positives. Consequently, our selection results are expected to be less sensitive but more stringent than those obtained from individual sequencing approaches. Both strategies capture complementary aspects of evolutionary change: individual‐level analyses detect selection correlated with measured traits, whereas pooled, drift‐calibrated genomic scans emphasise robust, replicable allele‐frequency shifts across the whole genome.

In the SNP‐level detection, we first computed absolute allele frequency difference (AFD) for every SNP in each population: $\mathrm{\Delta AF}={\mathrm{AF}}_{\text{final}}-{\mathrm{AF}}_{G1}$. For each population, the effective |ΔAF| threshold was set to the larger of the population‐specific 95^th^ percentile predicted under neutrality and the empirical control baseline (from previous section). A SNP was therefore considered to show significant shift only when |ΔAF| exceeded the relevant population‐specific threshold in both replicates within a treatment. Comparing ΔAF between ambient and hot, each SNP was assigned to one of four classes: (1) Background, |ΔAF| below the calibrated threshold in both regimes; (2) Global adaptation to pollinator, same‐signed shifts above threshold in both regimes. (3) Conditional adaptation, above threshold in only one regime; and (4) Antagonistic pleiotropy, opposite‐signed shifts above threshold in both, indicating opposite fitness effects across temperatures.

To compensate for the lack of variance‐normalising denominator of AFD, we then applied Hudson's *F*_ST_ to evaluate allele frequency divergence between each final‐generation replicate and the initial G1 population with R package poolfstat v.2.1.2. Hudson's formulation is well‐suited for pool‐sequencing designs, as it accounts for both within‐ and between‐population variance and is less biased by uneven sequencing depth than other *F*_ST_ metrics, which allows for more robust statistical comparisons across loci and replicates, especially when combining data with variable coverage or sampling error (Bhatia *et al*., 2013; Hivert *et al*., 2018).

To ensure biological relevance and replicate‐level consistency, we applied a conservative three‐step filtering strategy aligned with the recalibrated nulls. First, directional consistency: SNPs were required to show allele‐frequency shifts in the same direction across both replicates (i.e. both increasing or both decreasing relative to G1). Second, replicate concordance: |ΔAF| had to exceed the calibrated thresholds (above) in both replicates simultaneously. Finally, outlier thresholding: each replicate's *F*_ST_ had to exceed its population‐specific neutral 95^th^ percentile from the drift analysis. This approach adapts earlier strategies based on AFD (e.g. López *et al*., 2025), while leveraging the statistical advantages of F_ST_ for population‐level allele frequency data. The resulting SNP set provided a stringent but scalable foundation for identifying high‐confidence selection signals in downstream analyses.

Accurately accounting for linkage disequilibrium (LD) is essential when identifying selection signals across the genome. However, traditional LD estimation based on individual genotypes is not directly applicable to pool‐sequencing data. To address this, we used LDx (Feder *et al*., 2012), a tool specifically designed to estimate LD from pooled allele frequencies. Despite its adaptation for pool‐seq, LDx has known limitations in precision, particularly under experimental evolution conditions with limited generations and low sampling variance. To avoid prematurely filtering informative sites while still accounting for LD structure, we instead applied the local score approach (Fariello *et al*., 2017). This method retains the full SNP dataset while detecting clusters of moderately significant SNPs, which are likely to reflect underlying LD or selection on linked haplotypes. This strategy allows us to preserve signals that might be disrupted by hard LD pruning, while still incorporating genomic structure in a statistically informed way. Based on a recent benchmarking evaluation of selection detection methods in pool‐sequencing data (Vlachos *et al*., 2019) and to account for replicate structure, we implemented a top‐performing region‐level approach to identify signatures of selection: the Evolve‐and‐Resequence Cochran–Mantel–Haenszel (ER_CMH, MartaPelizzola/ACER package) (Spitzer *et al*., 2020). This method is well‐suited to detecting clusters of moderately differentiated SNPs, which may reflect soft sweeps or linked selection variants. This likelihood‐based test evaluates allele frequency shifts across replicates relative to the initial G1 pool while adjusting for drift and sampling variance. The G1 pool was duplicated across replicates to match the evolved population structure. Resulting *P*‐values were corrected with the Benjamini–Hochberg false discovery rate (FDR) procedure to make Manhattan plot.

To identify genomic regions with clusters of moderately significant SNPs, we applied the local score approach based on the Lindley process (Fariello *et al*., 2017). Raw *P*‐values from ER_CMH were converted to scores using –log_10_(*q*) − ξ (ξ = 1 for relaxed threshold and ξ = 2 for strict threshold). Cumulative Lindley scores were calculated along each chromosome. Significance thresholds were determined by fitting a Gumbel distribution to the maxima of 5000 resampled score trajectories across genomic windows of 10–70 kb. Chromosome‐specific thresholds were applied at the 5% significance level to define enriched genomic intervals.

### Functional annotation of selective genomic regions

To improve specificity and account for the short experimental timescale, we integrated the outputs from the SNP‐level and region‐level analyses and retained only regions that were independently significant in both *F*_ST_ significant and ER_CMH‐local score analyses. This yielded a conservative set of high‐confidence loci under selection. The final candidate genes, identified through relaxed selection filtering, were annotated using Bedtools intersect against gene models from the *Brassica rapa* FPsc v.1.3 reference genome (Phytozome release 12). Gene identifiers and functional descriptions were extracted using custom parsing scripts and matched to the official annotation database. For each treatment, we performed Gene Ontology (GO) enrichment using topGO (classic Fisher test), with all annotated genes as the background. We report both raw *P*‐values and Benjamini–Hochberg FDR–adjusted *P*‐values (FDR < 0.05), noting that only a small number of terms remained significant after FDR correction. Enrichment results were used to highlight biological processes that may underlie treatment‐specific adaptive responses.

## Results

### Overall genomic divergence patterns

We sequenced and analysed pooled genomic data from 17 populations, including the initial generation (G1) and 16 evolved populations spanning eight treatment combinations (2 temperatures × 4 pollination regimes with 2 replicates). After quality filtering and variant calling, we retained a total of 1787 629 high‐confidence bi‐allelic SNPs across all samples for downstream analyses.

**Fig. 1:**
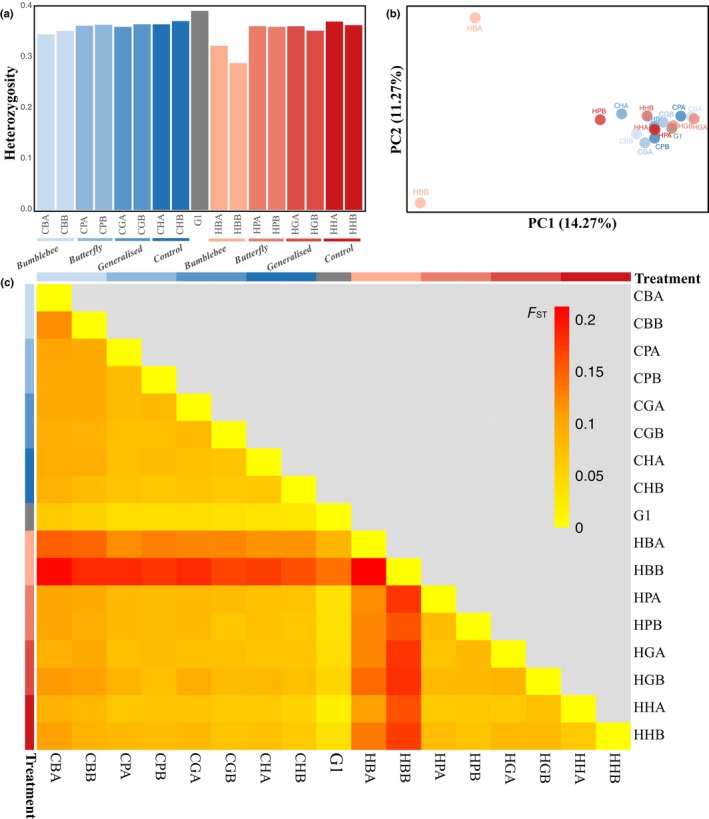
Genome‐wide patterns of diversity and population structure following experimental evolution. (a) Expected heterozygosity in initial (G1) and evolved populations under different temperature and pollination treatments. (b) Principal component analysis of genome‐wide allele frequencies, showing genetic divergence of evolved populations from G1 and clustering by treatment. (c) Pairwise Hudson's *F*_ST_ heatmap between all population pairs, reflecting genome‐wide genetic differentiation. Treatment codes follow the structure: first letter denotes temperature regime (C for ambient, H for hot), second letter indicates pollination regime (B: bumblebee (*Bombus terrestris*), P: butterfly (*Pieris rapae*), G: generalised, H: control (hand‐pollination)), and third letter indicates replicate (A or B). G1 samples are shown in dark grey; ambient treatments are shaded in blue, and hot treatments in red.

The G1 population exhibited a genome‐wide nucleotide diversity of π = 0.0086 per site (95% bootstrap CI: 0.00858–0.00863), calculated across all callable, nonrepetitive genomic sites. While this genome‐wide estimate reflects absolute genetic diversity across all sites (including invariant positions) and allows direct comparison with natural populations, subsequent analyses of population differentiation and heterozygosity were performed on the filtered SNP dataset. This SNP‐based dataset provides a robust basis for relative comparisons among treatments since all populations were aligned to the same reference genome and underwent identical variant calling and filtering procedures. Within this framework, compared to the G1 (expected heterozygosity = 0.39), all evolved populations exhibited reduced heterozygosity, with the most pronounced loss observed in hot environments with bumblebee‐pollination (HBA and HBB; *H*_e_ = 0.32 and 0.29, respectively; Fig. 1a).

PCA based on allele frequencies revealed variable levels of separation among treatments. Specifically, populations evolved under the hot bumblebee (HB) condition showed a distinct separation along the first principal component (PC1), which explained 16.5% of the variance, while other treatments clustered more closely together. Within the HB treatment, the two replicates (HBA and HBB) were further separated from each other along the second principal component (PC2), indicating both treatment‐specific and replicate‐level divergence (Fig. 1b). Pairwise *F*_ST_ values further supported these observations. The greatest genomic divergence from G1 was observed in the hot bumblebee treatment, with HBA and HBB showing *F*_ST_ values of 0.088 and 0.139, respectively. By contrast, other treatments displayed lower *F*_ST_ values, typically ranging from 0.021 to 0.063 (Fig. 1c).

We further calculated genome‐wide summary statistics, including nucleotide diversity (π), Watterson's θ and Tajima's *D*, to assess shifts in genetic variation across treatments. All evolved populations showed reduced nucleotide diversity relative to the initial pool, consistent with strong selection or genetic drift during the experimental evolution. The most pronounced reduction in π occurred in the hot bumblebee treatment, dropping from 0.318 (G1) to 0.194 and 0.204 in HBA and HBB, respectively (Table 1). Tajima's *D* values were consistently negative across treatments, with the strongest shift observed in the hot bumblebee populations (HBA: –2.874; HBB: −3.130). Given the known limitations of Pool‐seq data for estimating absolute values (Czech *et al*., 2024), we emphasise contrasts among treatments and directional consistency rather than interpreting absolute values.

**Table 1:** Relative genetic diversity metrics for initial and evolved populations.

Populations	Temperature	Pollinators	Rep	π_MEAN (SD)	θ_MEAN (SD)	Tajima's D_MEAN (SD)
CBA	Ambient	Bumblebee	A	0.219 (0.372)	0.905 (0.529)	−2.939 (2.268)
CBB	Ambient	Bumblebee	B	0.228 (0.319)	0.869 (0.485)	−2.758 (2.216)
CPA	Ambient	Butterfly	A	0.277 (0.408)	0.843 (0.453)	−2.549 (2.236)
CPB	Ambient	Butterfly	B	0.244 (0.368)	0.929 (0.508)	−2.879 (2.372)
CGA	Ambient	Generalised	A	0.297 (0.437)	0.919 (0.489)	−2.681 (2.25)
CGB	Ambient	Generalised	B	0.301 (0.434)	0.89 (0.481)	−2.478 (2.233)
CHA	Ambient	Control	A	0.291 (0.439)	0.944 (0.524)	−2.794 (2.373)
CHB	Ambient	Control	B	0.261 (0.373)	0.829 (0.43)	−2.571 (2.236)
G1				0.318 (0.414)	0.925 (0.47)	−2.621 (2.297)
HBA	Hot	Bumblebee	A	0.194 (0.328)	0.851 (0.504)	−2.874 (2.23)
HBB	Hot	Bumblebee	B	0.204 (0.399)	0.965 (0.589)	−3.13 (2.388)
HPA	Hot	Butterfly	A	0.322 (0.475)	0.937 (0.504)	−2.587 (2.235)
HPB	Hot	Butterfly	B	0.262 (0.461)	0.967 (0.523)	−3.052 (2.299)
HGA	Hot	Generalised	A	0.254 (0.355)	0.837 (0.442)	−2.549 (2.246)
HGB	Hot	Generalised	B	0.272 (0.408)	0.895 (0.516)	−2.572 (2.261)
HHA	Hot	Control	A	0.291 (0.398)	0.888 (0.472)	−2.543 (2.307)
HHB	Hot	Control	B	0.339 (0.457)	0.866 (0.469)	−2.435 (2.269)

Mean values (MEAN) and SD (SD) of nucleotide diversity (π), Watterson's theta (θ), and Tajima's *D* across the SNPs dataset for each population.

### Demography and drift impact genome‐wide divergence and the resolution of selection signals

Genome‐wide reductions in heterozygosity, elevated *F*_ST_, and negative Tajima's *D* in the bumblebee treatments coincide with realised small *N*_e_, whereas the control populations and other pollinator regimes retained considerably larger values. Neutral Wright–Fisher simulations parameterised by these trajectories reproduced the magnitude of genome‐wide drift expected under each demographic regime. The resulting 95^th^ percentiles of absolute allele‐frequency change (|ΔAF|) and Hudson's *F*_ST_ differed markedly among populations, with bumblebee regimes yielding the highest thresholds (|ΔAF|95 ≈ 0.34–0.35; *F*_ST_95 ≈ 0.55–0.57; Table S2). The empirical control baseline provided an additional benchmark for glasshouse‐specific variance (|ΔAF|95 = 0.32–0.36), and the final cut‐offs for each treatment were defined as the maximum between the neutral and empirical limits. For most populations, the empirical‐control distribution determined the effective threshold, whereas in bumblebee populations, the neutral expectation dominated owing to their small *N*_e_.

### Environmental treatments drive distinct genomic signatures of selection

**Fig. 2:**
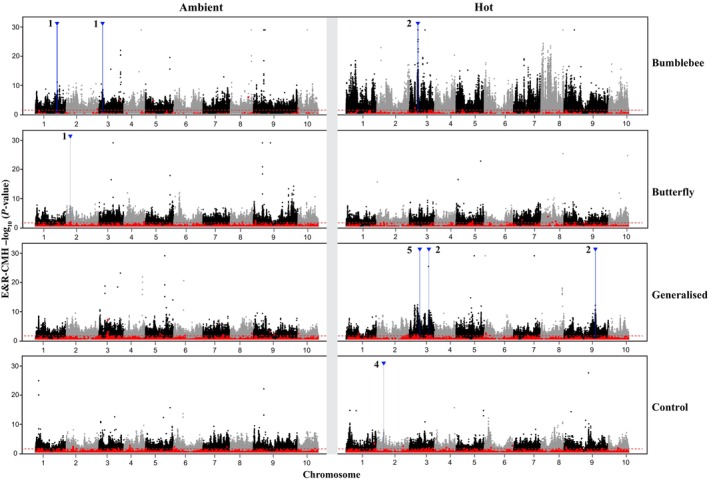
Genome‐wide single‐nucleotide polymorphism (SNP)‐level and local score regional selection signals across treatments. The basic Manhattan plots showed −log_10_(*P*‐values) from Evolve‐and‐Resequence Cochran–Mantel–Haenszel test (ER_CMH) across all 10 chromosomes, separated by black and grey. The horizontal panels from top to bottom represent pollination regimes: bumblebee, butterfly, generalised, and control, respectively. The vertical panels from left to right represent ambient and hot regimes, respectively. The black and grey dots represent all SNPs in different chromosomes. Red dashed lines indicate false discovery rate = 0.05 in ER_CMH. Red dots represent the SNPs that are significantly different from neutral drift simulations. Blue vertical lines and downward triangles highlight genomic regions where ER_CMH outlier overlap with the *F*_ST_ outliers, suggesting convergent signals of selection. The thickness of each vertical line reflects the physical length of the overlap between ER_CMH and local score‐significant regions (see Table 2, for detailed chromosome, coordinates and length). Numbers near each blue peak indicate the count of significant SNPs within each overlapping region. No selection region was shared across pollinator or temperature regimes.

The composite selection signals among *F*_ST_ outlier scan, ER_CMH test and local score analysis showed a highly treatment‐specific pattern (Table 2; Fig. 2). Explicitly, we first tested for common genomic regions selected across treatments grouped by pollinator identity (i.e. bumblebee, butterfly, generalised‐pollination across temperature regimes) and by temperature conditions (i.e. hot vs ambient across pollinator treatments), but found no universally shared genomic regions responding consistently across these broader ecological factors, highlighting the strong specificity of genomic responses to each unique combination of pollinator identity and temperature. Overlaps within pollinator identities or temperature regimes were also limited, further highlighting the context‐dependent nature of selection.

**Table 2:** Summary of convergent selection signals by treatment.

	*F*_ST_‐thresholding exceed drift SNPs	ER_CMH significant (FDR < 0.05) SNPs	Local score genomic region		
			Chromosome	Genomic regions (Mb)	Length (kb)
Ambient‐bumblebee	213	5311	A01	21.20–22.16	957
			A03	3.30–3.84	578
Hot‐bumblebee	2177	61 653	A03	7.88–8.70	882
Ambient‐butterfly	7913	2670	A02	4.1725–4.1733	0.8
Hot‐butterfly	3698	1039	–	–	–
Ambient‐generalised	6252	2076	–	–	–
Hot‐generalised	6160	6133	A03	10.00–10.40	396
			A03	19.14–19.44	300
			A09	31.33–31.98	649
Ambient‐control	11 665	798	–	–	–
Hot‐control	8599	1092	A02	6.69–6.78	82

For each temperature × pollination treatment, we report: *F*_ST_ outliers exceeding drift expectations, defined as single‐nucleotide polymorphisms (SNPs) whose *F*_ST_ values exceed the treatment‐specific neutral range derived from the realised effective population size (*N*_e_); ER_CMH significant SNPs (count of SNPs with False Discovery Rate < 0.05 in the Evolve‐and‐Resequence Cochran–Mantel–Haenszel (ER_CMH) test, representing replicated allele‐frequency shifts across replicates); Local‐score genomic regions, clustered SNPs forming genomic regions consistent with regional selection. Coordinates refer to the *Brassica rapa* L. FPsc v.1.3 assembly.

Because each analytical layer captures a different aspect of allele‐frequency change, we summarised all results in three datasets according to increasing stringency (Table 2): (1) *F*_ST_‐thresholding filters for shifts unlikely under drift, (2) ER_CMH identifies replicated allele‐frequency shifts, and (3) local score clusters adjacent SNPs with consistent signals into genomic intervals. Candidate intervals that satisfy all three criteria represent the strongest evidence for selection. Eight genomic intervals met all three criteria: two in the ambient‐bumblebee treatment, one in hot‐bumblebee, one in ambient‐butterfly, three in hot‐generalised and one in hot‐hand. All other treatments, including hot‐butterfly treatments, ambient‐generalised, and cold‐hand, showed no significant intervals after drift correction. These outcomes reflect differences in both allele‐frequency trajectories and realised demographics. In the hot‐generalised treatment, moderate *N*_e_ and consistent replicate shifts produced clear, drift‐filtered intervals. By contrast, bumblebee treatments showed large genome‐wide divergence but few drift‐exceeding loci, a consequence of the small realised *N*_e_ imposed by the pollination protocol. Hot‐butterfly treatment exhibited allele‐frequency changes that fell entirely within neutral ranges, resulting in no significant intervals.

**Fig. 3:**
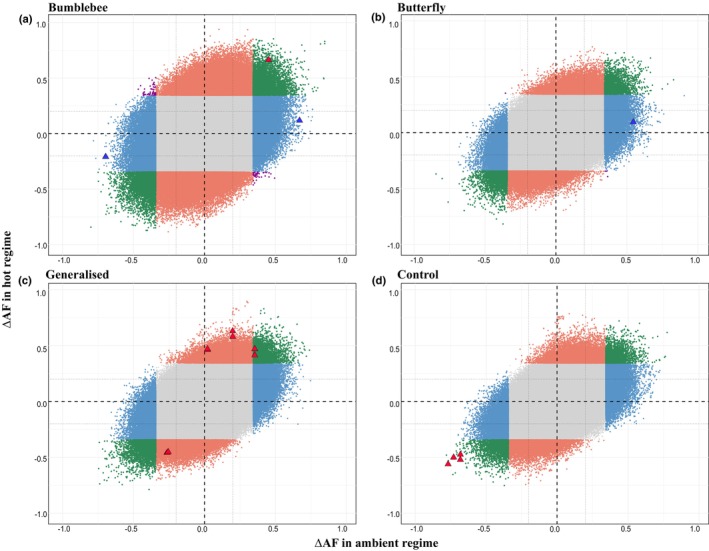
Temperature‐dependent *allele frequency change* reveals distinct patterns of genomic adaptation across pollinator treatments. Each panel shows allele frequency changes (ΔAF) between initial (G1) and evolved populations for different pollinator regimes: (a) bumblebee, (b) butterfly, (c) generalised (bumblebee + butterfly), (d) control. *X*‐axis represents ΔAF in ambient regime; *Y*‐axis represents ΔAF in hot regime. Black dashed lines indicate ΔAF = 0 (no change). Each dot represents a single‐nucleotide polymorphism (SNP). Classification: background SNPs (light grey dots) represent SNPs with |ΔAF| < threshold (0.345 for bumblebee, 0.343 for the rest) in both temperature regimes. Global adaptation SNPs to pollinator (green dots): |ΔAF| ≥ threshold in both temperature regimes. Conditional adaptation SNPs (blue dots for ambient‐specific, red dots for hot‐specific, respectively): |ΔAF| ≥ threshold in one condition but not the other, representing the loci responding specifically in temperature × pollinator interactions. Antagonistic pleiotropy SNPs (purple dots): |ΔAF| ≥ threshold in both temperature regimes but in opposite directions of change between temperatures, which are in upper‐left and lower‐right quadrant. This indicates loci where alleles beneficial in one temperature are detrimental in the other. Triangle overlays: SNPs overlapping with selection signals from other analyses (Evolve‐and‐Resequence Cochran–Mantel–Haenszel test and *F*_ST_), blue for ambient‐specific while red for hot‐specific, respectively.

Directional reversals across temperature regimes (antagonistic pleiotropy) occurred in few SNP‐level counts from *F*_ST_‐thresholding filters for shifts unlikely under drift (127 SNPs in bumblebee, five in butterfly, five in generalised, none in hand; Fig. 3; Table S3), but permutation analyses suggested these SNPs were within the threshold of neutral expectations. Thus, although allele‐frequency change was widespread, only a small subset of loci showed evidence for treatment‐specific selection, and these selective responses emerged under particular combinations of pollinator identity, temperature, and realised *N*_e_.

### Identification of candidate genes

To infer function of targets of selection, we annotated the stringiest genomic regions where independent scans converged: the regions supported by all three tests (Table 2). Across all treatments, 558 genes overlapped with these high‐confidence genomic regions (Table S4). The hot‐generalised and ambient‐bumblebee treatments contributed the largest genomic regions and greatest number of candidates (206 and 190 genes, respectively), whereas hot‐bumblebee treatments, despite exhibiting strong genome‐wide divergence, yielded a single large genomic region (882 kb) surpassing both neutral and empirical thresholds, consisting of 154 genes. Only small regions (0.8 kb and 82 kb consist of 2 and 6 genes, respectively) under selection in ambient‐butterfly and hot‐control treatments, respectively. And no regions were detected in hot‐butterfly or ambient‐generalised treatments.

**Fig. 4:**
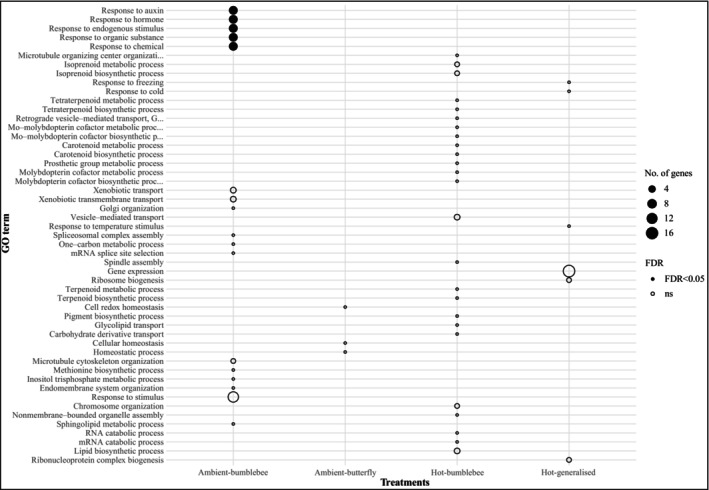
Gene Ontology (GO) Biological Process enrichment for genes located in selection‐supported genomic regions across treatments. GO enrichment (classic Fisher's exact test) was performed separately for each treatment using genes overlapping Evolve‐and‐Resequence Cochran–Mantel–Haenszel–local score candidate genomic regions. Circle size indicates the number of genes annotated for each GO term, and fill indicates whether terms remained significant after false discovery rate correction (FDR < 0.05). Only the ambient‐bumblebee treatment showed FDR‐significant enrichment, driven by auxin‐responsive processes. Hot‐bumblebee, hot‐generalised, and ambient‐butterfly treatments exhibited only nominally enriched terms (raw *P* < 0.05), reflecting secondary metabolism, membrane trafficking, temperature‐response, and cellular homeostasis, respectively. Full GO lists and associated gene sets are provided in Supporting Information Table S5.

GO enrichment yielded few FDR‐corrected terms overall (Table S5). Ambient‐bumblebee was the only treatment showing FDR‐significant enrichment, with five biological process terms, all linked to auxin and hormone responses. They are driven by a cluster of small auxin up‐regulated RNA (SAUR)‐like auxin‐responsive genes (Brapa.C00791/92/93/94/95/96/97/99). These results point to hormonal regulation and growth responses as the most coherent signature of selection in this treatment. Other treatments showed no FDR‐significant GO terms. However, several nominally enriched categories (raw *P* < 0.05) highlighted treatment‐specific tendencies: secondary‐metabolic and membrane‐trafficking processes in hot‐bumblebee populations, stress‐ and temperature‐response terms in the hot‐generalised treatment, and redox and cellular homeostasis in the butterfly treatments (Fig. 4). Given the small number of selected loci, we interpret nominal enrichments cautiously, but together with the candidate‐gene list they provide a functional scaffold for downstream hypotheses.

## Discussion

### Ecological interactions generate highly context‐dependent genomic responses

Our evolve‐and‐resequencing experiment revealed treatment‐specific genomic signatures shaped by interactions between biotic (pollinator identity) and abiotic (temperature) conditions. Overall, selection signals varied substantially across treatments, with no universal genomic regions consistently shared by all treatments or even within broader groupings based on either pollinator identity or temperature alone. Rather, genomic responses were distinctly contingent upon specific combinations of pollinator identity and temperature, underscoring the critical role of ecological interactions in shaping adaptive genomic trajectories.

### Standing genetic variation and demographic constraints set the evolutionary baseline

Adaptation in short‐term experimental evolution depends fundamentally on both the amount of standing genetic variation and the demographic conditions under which selection operates. Our experiment began with a genetically diverse starting population generated from 98 full‐sib families, reflected in a genome‐wide nucleotide diversity estimate π = 0.0086. This is closely matching values reported for natural *B. rapa* populations (π ≈ 0.0096; Franks *et al*., 2016). This confirms that our starting population retained natural levels of polymorphism, providing sufficient standing variation to respond to selection (Hermisson & Pennings, 2005, 2017; Barrett & Schluter, 2008).

The pollination treatments were designed to impose pollen limitation, a widespread feature of natural plant populations (Ashman *et al*., 2004; Knight *et al*., 2005). Bumblebees were given restricted foraging periods to maintain variation in male reproductive success, whereas butterflies naturally provide lower pollen transfer efficiency and work fewer flowers in one plant visit. As a consequence, only a subset of plants contributed seeds each generation (Traine *et al*., 2024), producing treatment‐specific realised effective population sizes. Such variation in *N*_e_ is expected in both controlled and natural systems, in which unequal reproductive success, phenological mismatch, and stochastic pollinator visitation routinely lower *N*_e_ relative to census size (Willi *et al*., 2007). These demographic filters shape the background variance in allele‐frequency change (Wright, 1931; Charlesworth, 2009) and therefore influence how readily selection can generate detectable genomic signals over six generations. Recognising these baseline constraints is essential: the genomic signatures we recover reflect not only the strength and direction of selection but also the opportunity for selection to overcome drift under each ecological scenario.

### Temperature modulates the strength, detectability, and genomic architecture of pollinator‐mediated selection

Temperature changed both the strength and the resolution of pollinator‐mediated selection. These thermal effects acted through shifts in pollinator behaviours, community composition, and realised effective population size. Pollinator taxa differ strongly in their thermal tolerance and behavioural responses to warming. In our system, bumblebee visitation remained stable, whereas butterfly visitation increased sharply at higher temperatures (Traine *et al*., 2024, 2025; Traine, 2025). This variation reflects patterns documented across natural communities (Creux *et al*., 2021; Moss & Evans, 2022), where warming reshapes interaction timing, foraging frequency, and network structure in species‐specific ways. Such heterogeneity alters the direction and strength of selection. Some plant–pollinator links weaken when thermal stress disrupts synchrony or reduces reward quality (Descamps *et al*., 2021). Other interactions intensify because warming promotes activity in certain insect groups (Rojas *et al*., 2024).

Bumblebee pollination has been shown to exert strong directional selection on *B. rapa*'s floral traits and lead to rapid evolution (Gervasi & Schiestl, 2017; Ramos & Schiestl, 2019; Dorey *et al*., 2024; Frachon & Schiestl, 2024). Our genomic results are consistent with strong bumblebee‐mediated selection, yet the extent to which these signals are detectable depends strongly on both temperature and resolution afforded by the sequencing approach. Compare to the ambient regime, the plants under warming yields a genome‐wide differentiation. Yet in the bumblebee‐only treatments, the small realised population size generated substantial genome‐wide drift. This inflated background noise allowed only a subset of truly selected loci to exceed our conservative, drift‐based thresholds. However, this reduced detectability does not necessarily indicate weak selection but rather reflects the limits of pool‐sequencing for resolving locus‐specific effects in the presence of strong drift. As suggested in the phenotypic analyses, Traine *et al*. (2024, 2025) show that bumblebees impose strong, multivariate selection across environments. By contrast, studies using individually sequenced plants (e.g. Dorey *et al*., 2024) can identify many more candidate loci because genotype–phenotype associations provide direct evidence of fitness effects, enabling detection of selection even when drift is expected to be substantial. Thus, the smaller number of high‐confidence regions in our bumblebee treatments reflects methodological stringency rather than weaker selection and should be interpreted as a conservative genomic footprint that remains after accounting for demographic noise and replicate consistency.

Even under these conservative criteria, a small subset of loci (127 SNPs) displayed opposite allele‐frequency shifts between temperatures. Although these counts did not exceed permutation‐based expectations, the pattern is qualitatively consistent with temperature‐dependent antagonistic pleiotropy. Such environmentally inverted allele effects are well‐documented. For example, HSP101 enhances thermotolerance but reduces fruit set in benign conditions (Qin *et al*., 2021), MAF2–5 modulates floral traits through temperature‐dependent splicing (Wiszniewski *et al*., 2022), and anthocyanin‐pathway genes produce attractive pigmentation at ambient temperatures but reduced colour under heat (Alcantud *et al*., 2023). These mechanisms illustrate how warming can reverse fitness consequences of the same allele and provide a plausible basis for the subtle opposing trajectories we observe. Importantly, phenotypic outcomes mirror this genomic interpretation: plants evolving under bumblebee‐pollination maintained fruit and seed production under heat, whereas hand‐pollinated controls suffered pronounced fertility loss in hot conditions (Traine *et al*., 2025). Thus, bumblebee visitation led to adaptive evolution buffering negative effects of warm temperature, even though demographic constraints limited our ability to detect the full genomic footprint of selection.

In the butterfly pollination treatment, the genomic response was strikingly weak despite strong directional phenotypic selection documented in the same experimental system. Phenotypic analyses showed that butterflies imposed clear positive directional selection on multiple floral and vegetative traits at both temperatures, with temperature shifting the specific trait targets (e.g. leaf number under heat) rather than reducing selection strength (Traine, 2025; Traine *et al*., 2025). Yet, despite this consistent phenotypic selection, no genomic regions exceeded our drift‐corrected thresholds in hot environments.

This disconnect suggests that selection in butterfly‐pollinated populations was diffuse, polygenic, or acting through regulatory pathways not detectable as strong allele‐frequency shifts over six generations. The dual role of *P. rapae* as both pollinator and herbivore likely amplified this complexity: Warming increased oviposition and larval feeding, creating a mixed mutualistic–antagonistic regime that can weaken or diversify genomic responses. Supporting this, previous work in *B. rapa* shows that herbivory can induce heritable epigenetic changes in floral traits without accompanying DNA‐sequence evolution (Kellenberger *et al*., 2016, 2018). Such mechanisms offer a plausible explanation for why phenotypic evolution was evident, but allele frequencies remained largely unchanged. Together, these results indicate that butterfly‐mediated selection was strong at the phenotypic level but not expressed as concentrated genomic sweeps, most likely because selection operated on many small‐effect loci, on epigenetic regulation, or on traits whose genetic bases were insufficiently large to exceed drift under the experimental timescale and population sizes (Schmid *et al*., 2018). Such nonparallel genomic responses despite parallel phenotypic evolution have been documented across diverse experimental evolution systems, including previous work in this *B. rapa* system (Frachon & Schiestl, 2024) and others spanning *Drosophila*, marine flies, *Arabidopsis*, and wild strawberry (Barghi *et al*., 2019; De Kort *et al*., 2022; Konečná *et al*., 2022; Fuhrmann *et al*., 2023).

In the generalised treatments, in which *B. rapa* interacted simultaneously with multiple mutualistic and antagonistic partners, warming reshaped the selective landscape in ways not observed in the single‐pollinator regimes. In our experiment, butterflies greatly increased their visitation rates under heat, with warming elevating total floral visits nearly fivefold in the generalised condition (Traine, 2025). This rise increased the number of seeds that reproduced each generation. Although it does not change *N*_e_ between temperature regimes, the larger *N*_e_ reduced drift relative to the bumblebee‐only treatment, in which pollen‐limitation controls restricted reproduction to a small subset of plants and drift overwhelmed locus‐specific signals. Importantly, this demographic shift clarifies why strong genomic signatures appeared in the hot‐generalised regime: the same bumblebee‐mediated selection that was present but masked in the bumblebee‐only treatments became detectable once drift was buffered by community‐level pollination.

This interpretation is consistent with the absence of clear selection peaks in butterfly‐only treatments, in which genomic responses were weak and likely diffuse across many small‐effect or regulatory loci. By contrast, under the community context, bumblebee‐imposed selection could rise above the reduced drift background and produce discrete genomic intervals. Phenotypic analyses from the same experimental system showed that warming also changed the traits under selection in these mixed communities – favouring, for example, increased leaf number and altered volatile emissions rather than classic pollinator attraction traits. The genomic intervals detected only in the hot‐generalised treatment align with these shifts and indicate that elevated temperature, increased butterfly activity, and intensified herbivory together created emergent and detectable selection pressures that were absent in ambient conditions and not predictable from single‐pollinator treatments alone.

Taken together, these results converge on a general principle: the genomic imprint of pollination is shaped not only by pollinator identity and temperature but also by the demographic consequences of their interaction behaviours. Bumblebees are capable of imposing strong directional selection, yet in single‐pollinator treatments the small realised *N*_e_ caused by pollen‐limitation controls allowed drift to dominate, masking most locus‐specific signatures. Only ecological contexts that maintain larger numbers of reproductive contributors, such as the community‐level treatments where butterfly visitation greatly increased *N*_e_, permitted bumblebee‐mediated selection to rise above background noise and become detectable in genomic data. Temperature further modified both the strength and detectability of selection by altering visitation rates, affecting herbivory pressure, and shifting how many plants contributed offspring each generation.

Across treatments, the genomic architecture of adaptation was therefore subtle, diffuse, and highly context‐dependent. This is consistent with theoretical and empirical expectations that rapid evolution in outcrossing plants often proceeds through polygenic and regulatory changes rather than single large‐effect loci (Yeaman, 2015; Hermisson & Pennings, 2017; Barghi *et al*., 2019; Schlötterer, 2023). This helps explain why phenotypic selection gradients were strong and repeatable across environments, whereas genomic signals were sharply contingent on *N*_e_, temperature, and community composition.

### Functional insights from candidate genomic regions

In the GO enrichment analysis, although only five GO terms exceeded both drift‐corrected and empirical control‐derived thresholds, the functional patterns emerging from these candidates provide insight into the molecular pathways that may underpin rapid adaptation in this system. The strongest signal came from ambient‐bumblebee treatments, which showed significant enrichment of auxin‐ and hormone‐responsive biological process terms. This signal was driven by a cluster of SAUR‐like auxin‐responsive genes, a gene family widely implicated in growth regulation, floral development, and hormone‐mediated responses to biotic and abiotic cues. Auxin signalling influences several pollinator‐relevant traits, including floral morphology, branching, and resource allocation, suggesting that even under relatively mild thermal conditions, bumblebee pollination can favour regulatory variants that tune developmental processes.

Although other treatments did not retain FDR‐significant GO enrichment, the nominally enriched terms reveal biologically meaningful patterns that connect our genomic signals to known phenotypic responses. In the hot‐bumblebee treatment, enriched categories were dominated by pathways involved in specialised secondary metabolism, including terpenoid and carotenoid biosynthesis, and membrane‐trafficking processes. These processes are directly relevant to floral volatile production and emission, which showed clear phenotypic shifts in the same experimental system (Traine *et al*., 2025). Among the candidate loci, geranyl diphosphate synthase 1 (GPPS1; Brara.C01749) is particularly notable. GPPS1 encodes geranyl diphosphate synthase, the rate‐limiting enzyme at the entry point of the monoterpene pathway that produces many key floral volatiles (Wang & Dixon, 2009; Muhlemann *et al*., 2014). Monoterpenes constitute major components of *Brassica* floral scent (Knudsen *et al*., 2006) and play important roles in mediating pollinator attraction (Farré‐Armengol *et al*., 2020). Even though GPPS1 did not surpass the strict FDR threshold, it consistently appeared among SNPs exceeding relaxed drift thresholds and nominal ER_CMH significance. This pattern is consistent with polygenic, small‐effect responses distributed across biosynthetic pathways rather than concentrated selective sweeps at single, large‐effect loci. The presence of GPPS1 within nominally enriched categories therefore provides mechanistic support for the phenotypic changes observed, indicating that warming and pollinator behaviour may shift selection towards the regulation and allocation of floral scent production, even when genome‐wide drift noise limits the detectability of individual loci. These connections illustrate how phenotype‐linked pathways can leave subtle genomic footprints under realistic ecological and demographic conditions.

In the hot‐generalised treatment, nominal enrichment of stress‐ and temperature‐response processes, together with signatures linked to translational capacity, suggests that plants under combined mutualistic and antagonistic pressure may rely on broad stress‐response pathways rather than specialised floral signalling modules. Butterfly‐pollinated populations showed the weakest enrichment, restricted to redox and cellular homeostasis, consistent with our finding that phenotypic shifts in this treatment likely arise through plasticity or regulatory changes that do not generate strong allele‐frequency divergence.

Taken together, these patterns indicate that rapid adaptation in this system acts primarily on small sets of regulatory and metabolic genes rather than broad genomic regions. The functional signatures are ecologically consistent: bumblebee‐mediated selection under benign thermal conditions favours hormonal and developmental regulators, while elevated temperature and community complexity shift selection towards stress physiology and resource allocation processes. Although the limited number of selected loci constrains functional inference, the overlap between candidate genes and biologically meaningful pathways provides a tractable foundation for future mechanistic work, including gene expression profiling, fine‐mapping, and functional assays.

### Broader implications for plant adaptation under environmental change

Our findings emphasise that predicting plant adaptation under climate change requires considering not only abiotic stress but also the ecological context in which reproduction occurs. Pollinator identity, pollinator behaviour, and temperature interact in nonadditive ways that shape both the strength and detectability of selection. Demography emerges as a central determinant of whether adaptive responses are captured at the genomic level, suggesting that population size fluctuations in natural systems may profoundly influence evolutionary trajectories. By integrating ecological observations, genomic analyses, and theoretical expectations, our study provides a mechanistic framework for understanding how rapid adaptation unfolds in complex environments. More broadly, these results highlight the importance of factorial and ecologically realistic experiments for forecasting evolutionary outcomes in a changing world.

## Competing interests

None declared.

## Author contributions

YD and FPS designed the research. YD performed the DNA extraction, statistical analysis, and wrote the manuscript. YD and FPS reviewed and edited the manuscript.

## Disclaimer

The New Phytologist Foundation remains neutral with regard to jurisdictional claims in maps and in any institutional affiliations.

## Supporting information


**Table S1** Realised effective population sizes (*N*_e_) across generations and final pool‐sequencing sample sizes for all experimental populations.
**Table S2** Drift‐based and empirical thresholds for allele‐frequency change and *F*_ST_.
**Table S3** Classification of SNPs by adaptive category based on final drift–control baseline thresholds.
**Table S4** Candidate genes located within high‐confidence selection intervals.
**Table S5** Gene Ontology biological process enrichment across treatments.
Please note: Wiley is not responsible for the content or functionality of any Supporting Information supplied by the authors. Any queries (other than missing material) should be directed to the *New Phytologist* Central Office.

## Data Availability

The raw sequence reads are available in the European Nucleotide Archive with project no. PRJEB106602 (https://www.ebi.ac.uk/ena/browser/view/PRJEB106602). All codes and scripts required to reproduce the analyses are available at https://github.com/dingyanqian/Brassica_rapa_pool_sequencing.git. Derived datasets were generated as part of the analysis workflow and can be regenerated from the raw data using the provided code.

## References

[nph70977-bib-0001] Alcantud R , Weiss J , Terry MI , Bernabé N , Verdú‐Navarro F , Fernández‐Breis JT , Egea‐Cortines M . 2023. Flower transcriptional response to long term hot and cold environments in *Antirrhinum majus* . Frontiers in Plant Science 14: 1120183. 36778675 10.3389/fpls.2023.1120183 PMC9911551

[nph70977-bib-0002] Anderson JT , Mitchell‐Olds T . 2011. Ecological genetics and genomics of plant defences: Evidence and approaches. Functional Ecology 25: 312–324. 21532968 10.1111/j.1365-2435.2010.01785.x PMC3082142

[nph70977-bib-0003] Ashman TL , Knight TM , Steets JA , Amarasekare P , Burd M , Campbell DR , Dudash MR , Johnston MO , Mazer SJ , Mitchell RJ *et al*. 2004. Pollen limitation of plant reproduction: Ecological and evolutionary causes and consequences. Ecology 85: 2408–2421.

[nph70977-bib-0004] Barghi N , Tobler R , Nolte V , Jakšić AM , Mallard F , Otte KA , Dolezal M , Taus T , Kofler R , Schlötterer C . 2019. Genetic redundancy fuels polygenic adaptation in *Drosophila* . PLoS Biology 17: e3000128. 30716062 10.1371/journal.pbio.3000128 PMC6375663

[nph70977-bib-0005] Barrett RD , Schluter D . 2008. Adaptation from standing genetic variation. Trends in Ecology & Evolution 23: 38–44. 18006185 10.1016/j.tree.2007.09.008

[nph70977-bib-0006] Bhatia G , Patterson N , Sankararaman S , Price AL . 2013. Estimating and interpreting F_ST_: the impact of rare variants. Genome Research 23: 1514–1521. 23861382 10.1101/gr.154831.113 PMC3759727

[nph70977-bib-0007] Bishop J , Jones HE , OSullivan DM , Potts SG . 2017. Elevated temperature drives a shift from selfing to outcrossing in the insect‐pollinated legume, faba bean (*Vicia faba*). Journal of Experimental Botany 68: 2055–2063. 27927999 10.1093/jxb/erw430 PMC5429019

[nph70977-bib-0008] Bradshaw HD , Schemske DW . 2003. Allele substitution at a flower colour locus produces a pollinator shift in monkeyflowers. Nature 426: 176–178. 14614505 10.1038/nature02106

[nph70977-bib-0009] Cantila AY , Chen S , Siddique KHM , Cowling WA . 2024. Heat shock responsive genes in Brassicaceae: genome‐wide identification, phylogeny, and evolutionary associations within and between genera. Genome 67: 464–481. 39412080 10.1139/gen-2024-0061

[nph70977-bib-0010] Caruso CM , Eisen KE , Martin RA , Sletvold N . 2019. A meta‐analysis of the agents of selection on floral traits. Evolution 73: 4–14. 30411337 10.1111/evo.13639

[nph70977-bib-0011] Charlesworth B . 2009. Effective population size and patterns of molecular evolution and variation. Nature Reviews Genetics 10: 195–205. 10.1038/nrg2526 19204717

[nph70977-bib-0012] Cohen SP , Leach JE . 2020. High temperature‐induced plant disease susceptibility: more than the sum of its parts. Current Opinion in Plant Biology 56: 235–241. 32321671 10.1016/j.pbi.2020.02.008

[nph70977-bib-0013] Creux NM , Brown RI , Garner AG , Saeed S , Scher CL , Holalu SV , Yang D , Maloof JN , Blackman BK , Harmer SL . 2021. Flower orientation influences floral temperature, pollinator visits and plant fitness. New Phytologist 232: 868–879. 34318484 10.1111/nph.17627

[nph70977-bib-0014] Czech L , Spence JP , Espósito‐Alonso M . 2024. grenedalf: population genetic statistics for the next generation of pool sequencing. Bioinformatics 40: btae508. 39185959 10.1093/bioinformatics/btae508 PMC11357794

[nph70977-bib-0015] Danecek P , Auton A , Abecasis G , Albers CA , Banks E , DePristo MA , Handsaker RE , Lunter G , Marth GT , Sherry ST *et al*. 2011. The variant call format and VCFtools . Bioinformatics 27: 2156–2158. 21653522 10.1093/bioinformatics/btr330 PMC3137218

[nph70977-bib-0016] De Kort H , Toivainen T , Van Nieuwerburgh F , Andrés J , Hytönen TP , Honnay O . 2022. Signatures of polygenic adaptation align with genome‐wide methylation patterns in wild strawberry plants. New Phytologist 235: 1501–1514. 35575945 10.1111/nph.18225

[nph70977-bib-0017] De‐la‐Cruz IM , Batsleer F , Bonte D , Diller C , Hytönen T , Muola A , Osorio S , Posé D , Vandegehuchte ML , Stenberg JA . 2022. Evolutionary ecology of plant–arthropod interactions in light of the “Omics” sciences: a broad guide. Frontiers in Plant Science 13: 808427. 35548276 10.3389/fpls.2022.808427 PMC9084618

[nph70977-bib-0018] Depristo MA , Banks E , Poplin R , Garimella KV , Maguire JR , Hartl C , Philippakis AA , Del Angel G , Rivas MA , Hanna M *et al*. 2011. A framework for variation discovery and genotyping using next‐generation DNA sequencing data. Nature Genetics 43: 491–501. 21478889 10.1038/ng.806 PMC3083463

[nph70977-bib-0019] Desaint H , Aoun N , Deslandes L , Vailleau F , Roux F , Berthomé R . 2021. Fight hard or die trying: when plants face pathogens under heat stress. New Phytologist 229: 712–734. 32981118 10.1111/nph.16965

[nph70977-bib-0020] Descamps C , Jambrek A , Quinet M , Jacquemart AL . 2021. Warm temperatures reduce flower attractiveness and bumblebee foraging. Insects 12: 493. 34070688 10.3390/insects12060493 PMC8226554

[nph70977-bib-0021] Dixit S , Sivalingam PN , Baskaran RKM , Senthil‐Kumar M , Ghosh PK . 2024. Plant responses to concurrent abiotic and biotic stress: unravelling physiological and morphological mechanisms. Plant Physiology Reports 29: 6–17.

[nph70977-bib-0022] Dorey T , Frachon L , Rieseberg LH , Kreiner JM , Schiestl FP . 2024. Biotic interactions promote local adaptation to soil in plants. Nature Communications 15: 1–12. 10.1038/s41467-024-49383-x PMC11189560 38890322

[nph70977-bib-0023] Dorey T , Schiestl FP . 2024. Bee‐pollination promotes rapid divergent evolution in plants growing in different soils. Nature Communications 15: 2703. 10.1038/s41467-024-46841-4 PMC10973342 38538597

[nph70977-bib-0024] Fariello MI , Boitard S , Mercier S , Robelin D , Faraut T , Arnould C , Recoquillay J , Bouchez O , Salin G , Dehais P *et al*. 2017. Accounting for linkage disequilibrium in genome scans for selection without individual genotypes: The local score approach. Molecular Ecology 26: 3700–3714. 28394503 10.1111/mec.14141

[nph70977-bib-0025] Farré‐Armengol G , Fernández‐Martínez M , Filella I , Junker RR , Peñuelas J . 2020. Deciphering the biotic and climatic factors that influence floral scents: a systematic review of floral volatile emissions. Frontiers in Plant Science 11: 1154. 32849712 10.3389/fpls.2020.01154 PMC7412988

[nph70977-bib-0026] Feder AF , Petrov DA , Bergland AO . 2012. LDx: Estimation of linkage disequilibrium from high‐throughput pooled resequencing data. PLoS ONE 7: e48588. 23152785 10.1371/journal.pone.0048588 PMC3494690

[nph70977-bib-0027] Federal Office of Meterology and Climatology Meteo Swiss . 2024. Climate change.

[nph70977-bib-0028] Fenster CB , Armbruster WS , Wilson P , Dudash MR , Thomson JD . 2004. Pollination syndromes and floral specialization. Annual Review of Ecology, Evolution, and Systematics 35: 375–403.

[nph70977-bib-0029] Figueira T , Frachon L , Schiestl FP . 2025. Bumblebee pollination and herbivory alter genomic adaptation of plants to soil. Molecular Ecology 34: e17811. 40485592 10.1111/mec.17811

[nph70977-bib-0030] Fischer AM , Strassmann KM , Croci‐Maspoli M , Hama AM , Knutti R , Kotlarski S , Schär C , Schnadt Poberaj C , Ban N , Bavay M *et al*. 2022. Climate scenarios for Switzerland CH2018 – approach and implications. Climate Services 26: 100288.

[nph70977-bib-0031] Frachon L , Arrigo L , Rusman Q , Poveda L , Qi W , Scopece G , Schiestl FP . 2023. Putative signals of generalist plant species adaptation to local pollinator communities and abiotic factors. Molecular Biology and Evolution 40: 1–15. 10.1093/molbev/msad036 PMC10015620 36795638

[nph70977-bib-0032] Frachon L , Schiestl FP . 2024. Rapid genomic evolution in *Brassica rapa* with bumblebee selection in experimental evolution. BMC Ecology and Evolution 24: 1–11. 38195402 10.1186/s12862-023-02194-y PMC10775529

[nph70977-bib-0033] Franks SJ , Kane NC , O'Hara NB , Tittes S , Rest JS . 2016. Rapid genome‐wide evolution in *Brassica rapa* populations following drought revealed by sequencing of ancestral and descendant gene pools. Molecular Ecology 25: 3622–3631. 27072809 10.1111/mec.13615 PMC4963267

[nph70977-bib-0034] Fuhrmann N , Prakash C , Kaiser TS . 2023. Polygenic adaptation from standing genetic variation allows rapid ecotype formation. eLife 12: e82824. 36852484 10.7554/eLife.82824 PMC9977305

[nph70977-bib-0035] Gautier M , Coronado‐Zamora M , Vitalis R . 2024. Estimating hierarchical *F*‐statistics from Pool‐Seq data. *bioRxiv*. doi: 10.1101/2024.11.22.624688.

[nph70977-bib-0036] Gautier M , Vitalis R , Flori L , Estoup A . 2022. F‐statistics estimation and admixture graph construction with Pool‐Seq or allele count data using the R package poolfstat . Molecular Ecology Resources 22: 1394–1416. 34837462 10.1111/1755-0998.13557

[nph70977-bib-0037] Gérard M , Gardelin E , Lehmann P , Roberts KT , Sepúlveda‐Rodríguez G , Sisquella C , Baird E . 2024. Experimental elevated temperature affects bumblebee foraging and flight speed. Proceedings of the Royal Society B: Biological Sciences 291: 20241598. 10.1098/rspb.2024.1598 PMC11521611 39471861

[nph70977-bib-0038] Gervasi DDL , Schiestl FP . 2017. Real‐time divergent evolution in plants driven by pollinators. Nature Communications 8: 14691. 10.1038/ncomms14691 PMC5424062 28291771

[nph70977-bib-0039] Gramlich S , Liu X , Favre A , Buerkle CA , Karrenberg S . 2022. A polygenic architecture with habitat‐dependent effects underlies ecological differentiation in *Silene* . New Phytologist 235: 1641–1652. 35586969 10.1111/nph.18260 PMC9544174

[nph70977-bib-0040] Grant V . 1949. Pollination systems as isolating mechanisms in angiosperms. Evolution 3: 82–97. 18115119 10.1111/j.1558-5646.1949.tb00007.x

[nph70977-bib-0041] Hermisson J , Pennings PS . 2005. Soft sweeps: molecular population genetics of adaptation from standing genetic variation. Genetics 169: 2335–2352. 15716498 10.1534/genetics.104.036947 PMC1449620

[nph70977-bib-0042] Hermisson J , Pennings PS . 2017. Soft sweeps and beyond: understanding the patterns and probabilities of selection footprints under rapid adaptation. Methods in Ecology and Evolution 8: 700–716.

[nph70977-bib-0043] Hivert V , Leblois R , Petit EJ , Gautier M , Vitalis R . 2018. Measuring genetic differentiation from pool‐seq data. Genetics 210: 315–330. 30061425 10.1534/genetics.118.300900 PMC6116966

[nph70977-bib-0044] Jagtap AB , Yadav IS , Vikal Y , Praba UP , Kaur N , Gill AS , Johal GS . 2023. Transcriptional dynamics of maize leaves, pollens and ovules to gain insights into heat stress‐related responses. Frontiers in Plant Science 14: 1117136. 36875566 10.3389/fpls.2023.1117136 PMC9975602

[nph70977-bib-0045] Jombart T . 2008. adegenet: a R package for the multivariate analysis of genetic markers. Bioinformatics 24: 1403–1405. 18397895 10.1093/bioinformatics/btn129

[nph70977-bib-0046] Jombart T , Ahmed I . 2011. adegenet 1.3‐1: new tools for the analysis of genome‐wide SNP data. Bioinformatics 27: 3070–3071. 21926124 10.1093/bioinformatics/btr521 PMC3198581

[nph70977-bib-0047] Kellenberger RT , Desurmont GA , Schlüter PM , Schiestl FP . 2018. *Trans*‐generational inheritance of herbivory‐induced phenotypic changes in *Brassica rapa* . Scientific Reports 8: 3536. 29476119 10.1038/s41598-018-21880-2 PMC5824794

[nph70977-bib-0048] Kellenberger RT , Schlüter PM , Schiestl FP . 2016. Herbivore‐induced DNA demethylation changes floral signalling and attractiveness to pollinators in *Brassica rapa* . PLoS ONE 11: e0166646. 27870873 10.1371/journal.pone.0166646 PMC5117703

[nph70977-bib-0049] Khan S , Jaral S , Kumari P , Verma S . 2024. Effect of elevated temperature on the rate of flowering anthesis and seed set in *Olea ferruginea* . Ecological Frontiers 44: 282–288.

[nph70977-bib-0050] Knauer AC , Schiestl FP . 2017. The effect of pollinators and herbivores on selection for floral signals: a case study in *Brassica rapa* . Evolutionary Ecology 31: 285–304.

[nph70977-bib-0051] Knight TM , Steets JA , Vamosi JC , Mazer SJ , Burd M , Campbell DR , Dudash MR , Johnston MO , Mitchell RJ , Ashman TL . 2005. Pollen limitation of plant reproduction: pattern and process. Annual Review of Ecology, Evolution, and Systematics 36: 467–497.

[nph70977-bib-0052] Knudsen JT , Eriksson R , Gershenzon J , Ståhl B . 2006. Diversity and distribution of floral scent. The Botanical Review 72: 1–20.

[nph70977-bib-0053] Kofler R , Orozco‐terWengel P , De Maio N , Pandey RV , Nolte V , Futschik A , Kosiol C , Schlötterer C . 2011a. PoPoolation: a toolbox for population genetic analysis of next generation sequencing data from pooled individuals. PLoS ONE 6: e15925. 21253599 10.1371/journal.pone.0015925 PMC3017084

[nph70977-bib-0054] Kofler R , Pandey RV , Schlötterer C . 2011b. PoPoolation2: identifying differentiation between populations using sequencing of pooled DNA samples (Pool‐Seq). Bioinformatics 27: 3435–3436. 22025480 10.1093/bioinformatics/btr589 PMC3232374

[nph70977-bib-0055] Kofler XV , Grossniklaus U , Schiestl FP , Frachon L . 2024. Uncovering genes involved in pollinator‐driven mating system shifts and selfing syndrome evolution in *Brassica rapa* . New Phytologist 243: 1220–1230. 38853408 10.1111/nph.19880

[nph70977-bib-0056] Konečná V , Šustr M , Požárová D , Čertner M , Krejčová A , Tylová E , Kolář F . 2022. Genomic basis and phenotypic manifestation of (non‐)parallel serpentine adaptation in *Arabidopsis arenosa* . Evolution 76: 2315–2331. 35950324 10.1111/evo.14593

[nph70977-bib-0057] Lasky JR , Des Marais DL , Lowry DB , Povolotskaya I , McKay JK , Richards JH , Keitt TH , Juenger TE . 2014. Natural variation in abiotic stress responsive gene expression and local adaptation to climate in *Arabidopsis thaliana* . Molecular Biology and Evolution 31: 2283–2296. 24850899 10.1093/molbev/msu170 PMC4137704

[nph70977-bib-0058] López ME , Ozerov M , Pukk L , Noreikiene K , Gross R , Vasemägi A . 2025. Dynamic outlier slicing allows broader exploration of adaptive divergence: a comparison of individual genome and pool‐seq data linked to humic adaptation in perch. Molecular Ecology 34: e17659. 39846218 10.1111/mec.17659 PMC11815547

[nph70977-bib-0059] de Manincor N , Fisogni A , Rafferty NE . 2023. Warming of experimental plant–pollinator communities advances phenologies, alters traits, reduces interactions and depresses reproduction. Ecology Letters 26: 323–334. 36592334 10.1111/ele.14158 PMC10107705

[nph70977-bib-0060] McKenna A , Hanna M , Banks E , Sivachenko A , Cibulskis K , Kernytsky A , Garimella K , Altshuler D , Gabriel S , Daly M *et al*. 2010. The genome analysis toolkit: a MapReduce framework for analysing next‐generation DNA sequencing data. Genome Research 20: 1297–1303. 20644199 10.1101/gr.107524.110 PMC2928508

[nph70977-bib-0061] Migicovsky Z , Yao Y , Kovalchuk I . 2014. Transgenerational phenotypic and epigenetic changes in response to heat stress in *Arabidopsis thaliana* . Plant Signaling & Behavior 9: e27971. 24513700 10.4161/psb.27971 PMC4091214

[nph70977-bib-0062] Minadakis N , Kaderli L , Horvath R , Bourgeois Y , Xu W , Thieme M , Woods DP , Roulin AC . 2024. Polygenic architecture of flowering time and its relationship with local environments in the grass *Brachypodium distachyon* . Genetics 227: iyae042. 38504651 10.1093/genetics/iyae042 PMC11075549

[nph70977-bib-0063] Moss ED , Evans DM . 2022. Experimental climate warming reduces floral resources and alters insect visitation and wildflower seed set in a cereal Agro‐ecosystem. Frontiers in Plant Science 13: 826205. 35283885 10.3389/fpls.2022.826205 PMC8905351

[nph70977-bib-0064] Muhlemann JK , Klempien A , Dudareva N . 2014. Floral volatiles: from biosynthesis to function. Plant, Cell & Environment 37: 1936–1949. 10.1111/pce.12314 24588567

[nph70977-bib-0065] Mukherjee K , Dubovskiy I , Grizanova E , Lehmann R , Vilcinskas A . 2019. Epigenetic mechanisms mediate the experimental evolution of resistance against parasitic fungi in the greater wax moth *Galleria mellonella* . Scientific Reports 9: 1–11. 30733453 10.1038/s41598-018-36829-8 PMC6367475

[nph70977-bib-0066] Naumchik M , Youngsteadt E . 2023. Larger pollen loads increase risk of heat stress in foraging bumblebees. Biology Letters 19: 20220581. 37194258 10.1098/rsbl.2022.0581 PMC10189305

[nph70977-bib-0067] Nawaz M , Sun J , Shabbir S , Khattak WA , Ren G , Nie X , Bo Y , Javed Q , Du D , Sonne C . 2023. A review of plants strategies to resist biotic and abiotic environmental stressors. Science of the Total Environment 900: 165832. 37524179 10.1016/j.scitotenv.2023.165832

[nph70977-bib-0068] Qin F , Yu B , Li W . 2021. Heat shock protein 101 (HSP101) promotes flowering under nonstress conditions. Plant Physiology 186: 407–419. 33561259 10.1093/plphys/kiab052 PMC8154077

[nph70977-bib-0069] R Core Team . 2023. R: a language and environment for statistical computing. Vienna, Austria: R Foundation for Statistical Computing. https://www.R‐project.org/.

[nph70977-bib-0070] Ramos SE , Schiestl FP . 2019. Rapid plant evolution driven by the interaction of pollination and herbivory. Science 364: 193–196. 30975889 10.1126/science.aav6962

[nph70977-bib-0071] Rojas SA , Escobedo VM , González‐Teuber M . 2024. Impacts of increased temperatures on floral rewards and pollinator interactions: a meta‐analysis. Frontiers in Plant Science 15: 1448070. 39582623 10.3389/fpls.2024.1448070 PMC11581868

[nph70977-bib-0072] Rusman Q , Lucas‐Barbosa D , Hassan K , Poelman EH . 2020. Plant ontogeny determines strength and associated plant fitness consequences of plant‐mediated interactions between herbivores and flower visitors. Journal of Ecology 108: 1046–1060. 32421019 10.1111/1365-2745.13370 PMC7217261

[nph70977-bib-0073] Rusman Q , Lucas‐Barbosa D , Poelman EH . 2018. Dealing with mutualists and antagonists: Specificity of plant‐mediated interactions between herbivores and flower visitors, and consequences for plant fitness. Functional Ecology 32: 1022–1035.

[nph70977-bib-0074] Rusman Q , Traine J , Schiestl FP . 2025. Elevated temperature diminishes reciprocal selection in an experimental plant–pollinator–herbivore system. Ecology Letters 28: e70060. 39805583 10.1111/ele.70060

[nph70977-bib-0075] Sato H , Mizoi J , Shinozaki K , Yamaguchi‐Shinozaki K . 2024. Complex plant responses to drought and heat stress under climate change. The Plant Journal 117: 1873–1892. 38168757 10.1111/tpj.16612

[nph70977-bib-0076] Scaven VL , Rafferty NE . 2013. Physiological effects of climate warming on flowering plants and insect pollinators and potential consequences for their interactions. Current Zoology 59: 418–426. 24009624 10.1093/czoolo/59.3.418 PMC3761068

[nph70977-bib-0077] Schiestl FP , Balmer A , Gervasi DD . 2018. Real‐time evolution supports a unique trajectory for generalised pollination. Evolution 72: 2653–2668. 30257033 10.1111/evo.13611

[nph70977-bib-0078] Schlötterer C . 2023. How predictable is adaptation from standing genetic variation? Experimental evolution in *Drosophila* highlights the central role of redundancy and linkage disequilibrium. Philosophical Transactions of the Royal Society of London. Series B: Biological Sciences 378: 20220046. 37004724 10.1098/rstb.2022.0046 PMC10067264

[nph70977-bib-0079] Schmid MW , Heichinger C , Coman Schmid D , Guthörl D , Gagliardini V , Bruggmann R , Aluri S , Aquino C , Schmid B , Turnbull LA *et al*. 2018. Contribution of epigenetic variation to adaptation in *Arabidopsis* . Nature Communications 9: 4446. 10.1038/s41467-018-06932-5 PMC6202389 30361538

[nph70977-bib-0080] Sletvold N , Moritz KK , Ågren J . 2015. Additive effects of pollinators and herbivores result in both conflicting and reinforcing selection on floral traits. Ecology 96: 214–221. 26236906 10.1890/14-0119.1

[nph70977-bib-0081] Spitzer K , Pelizzola M , Futschik A . 2020. Modifying the Chi‐square and the CMH test for population genetic inference: adapting to overdispersion. The Annals of Applied Statistics 14: 202–220.

[nph70977-bib-0082] Streisfeld MA , Rausher MD . 2009. Altered *trans*‐regulatory control of gene expression in multiple anthocyanin genes contributes to adaptive flower colour evolution in *Mimulus aurantiacus* . Molecular Biology and Evolution 26: 433–444. 19029190 10.1093/molbev/msn268

[nph70977-bib-0083] Traine J . 2025. The effects of warming and plant‐insect interactions on plant evolution . Dissertation, University of Zurich. doi: 10.5167/uzh-278129.

[nph70977-bib-0084] Traine J , Rusman Q , Schiestl FP . 2024. Too hot to handle: temperature‐induced plasticity influences pollinator behaviour and plant fitness. New Phytologist 243: 1571–1585. 38922897 10.1111/nph.19918

[nph70977-bib-0085] Traine J , Rusman Q , Schiestl FP . 2025. Adaptive evolution can mitigate the negative effects of temperature stress on plant‐pollinator interactions. New Phytologist 249: 554–568. 41204793 10.1111/nph.70705 PMC12676098

[nph70977-bib-0086] Van der Auwera GA , Carneiro MO , Hartl C , Poplin R , del Angel G , Levy‐Moonshine A , Jordan T , Shakir K , Roazen D , Thibault J *et al*. 2013. From FastQ data to high‐confidence variant calls: The genome analysis toolkit best practices pipeline. Current Protocols in Bioinformatics 43: 11.10.1–11.10.33. 10.1002/0471250953.bi1110s43 PMC4243306 25431634

[nph70977-bib-0087] Vasimuddin M , Misra S , Li H , Aluru S . 2019. Efficient architecture‐aware acceleration of BWA‐MEM for multicore systems. IEEE international parallel and distributed processing symposium (IPDPS), Rio de Janeiro, Brazil: IEEE, 314–324.

[nph70977-bib-0088] Vlachos C , Burny C , Pelizzola M , Borges R , Futschik A , Kofler R , Schlötterer C . 2019. Benchmarking software tools for detecting and quantifying selection in evolve and resequencing studies. Genome Biology 20: 1–11. 31416462 10.1186/s13059-019-1770-8 PMC6694636

[nph70977-bib-0089] Walters J , Zavalnitskaya J , Isaacs R , Szendrei Z . 2022. Heat of the moment: extreme heat poses a risk to bee–plant interactions and crop yields. Current Opinion in Insect Science 52: 100927. 35500861 10.1016/j.cois.2022.100927

[nph70977-bib-0090] Wang G , Dixon RA . 2009. Heterodimeric geranyl (geranyl) diphosphate synthase from hop (*Humulus lupulus*) and the evolution of monoterpene biosynthesis. Proceedings of the National Academy of Sciences, USA 106: 9914–9919. 10.1073/pnas.0904069106 PMC2701037 19482937

[nph70977-bib-0091] Wenzell KE , Neequaye M , Paajanen P , Hill L , Brett P , Byers KJRP . 2025. Within‐species floral evolution reveals convergence in adaptive walks during incipient pollinator shift. Nature Communications 16: 2721. 10.1038/s41467-025-57639-3 PMC11923230 40108138

[nph70977-bib-0092] Willi Y , Van Buskirk J , Schmid B , Fischer M . 2007. Genetic isolation of fragmented populations is exacerbated by drift and selection. Journal of Evolutionary Biology 20: 534–542. 17305819 10.1111/j.1420-9101.2006.01263.x

[nph70977-bib-0093] Wiszniewski A , Uberegui E , Messer M , Sultanova G , Borghi M , Duarte GT , Vicente R , Sageman‐Furnas K , Fernie AR , Nikoloski Z *et al*. 2022. Temperature‐mediated flower size plasticity in *Arabidopsis* . IScience 25: 105411. 36388994 10.1016/j.isci.2022.105411 PMC9646949

[nph70977-bib-0094] Wright S . 1931. Evolution in Mendelian populations. Genetics 16: 97–159. 17246615 10.1093/genetics/16.2.97 PMC1201091

[nph70977-bib-0095] Yadav NS , Titov V , Ayemere I , Byeon B , Ilnytskyy Y , Kovalchuk I . 2022. Multigenerational exposure to heat stress induces phenotypic resilience, and genetic and epigenetic variations in *Arabidopsis thaliana* offspring. Frontiers in Plant Science 13: 728167. 35419019 10.3389/fpls.2022.728167 PMC8996174

[nph70977-bib-0096] Yeaman S . 2015. Local adaptation by alleles of small effect. American Naturalist 186: S74–S89. 10.1086/682405 26656219

[nph70977-bib-0097] Zan Y , Carlborg Ö . 2019. A polygenic genetic architecture of flowering time in the worldwide *Arabidopsis thaliana* population. Molecular Biology and Evolution 36: 141–154. 30388255 10.1093/molbev/msy203

[nph70977-bib-0098] Zhu JK . 2016. Abiotic stress signalling and responses in plants. Cell 167: 313–324. 27716505 10.1016/j.cell.2016.08.029 PMC5104190

